# Effects of Non-Surgical Periodontal Therapy on Gingival Crevicular Fluid Apelin Isoforms and Oxidative Stress Markers in Periodontitis

**DOI:** 10.3390/diagnostics16152484

**Published:** 2026-08-06

**Authors:** Gozde Nur Aydogan, Hatice Yemenoglu, Oguz Kose, Semih Alperen Bostan, Sibel Mataraci Karakas, Adnan Yilmaz

**Affiliations:** 1 Department of Periodontology, Faculty of Dentistry, Recep Tayyip Erdoğan University, Rize 53020, Turkey; gozdenur.aydogan@erdogan.edu.tr (G.N.A.); oguz.kose@erdogan.edu.tr (O.K.); semihalperen.bostan@erdogan.edu.tr (S.A.B.); 2 Department of Medical Biochemistry, Faculty of Medicine, Recep Tayyip Erdoğan University, Rize 53020, Turkey; sibel.karakas@erdogan.edu.tr (S.M.K.); adnan.yilmaz@erdogan.edu.tr (A.Y.)

**Keywords:** apelin, gingival crevicular fluid, oxidative stress, periodontitis, periodontal therapy

## Abstract

**Background/Objectives:** Periodontitis is a chronic inflammatory disease in which host response and oxidative stress contribute to periodontal tissue destruction. Apelin isoforms may be involved in inflammatory regulation, tissue remodeling, and oxidative balance. This study evaluated changes in gingival crevicular fluid (GCF) apelin-13, apelin-36, total antioxidant status (TAS), total oxidant status (TOS), and oxidative stress index (OSI) following non-surgical periodontal therapy (NSPT) in systemically healthy individuals with stage I–II or stage III–IV periodontitis. **Methods:** A total of 61 systemically healthy individuals with periodontitis were included and divided into two groups: stage I–II periodontitis (n = 31) and stage III–IV periodontitis (n = 30). Clinical periodontal parameters and GCF samples were obtained at baseline and at 1, 3, and 6 months after NSPT. Apelin-13 and apelin-36 levels were measured by enzyme-linked immunosorbent assay, while TAS and TOS levels were determined using commercial assay kits. OSI was calculated as the percentage ratio of TOS to TAS. The study was retrospectively registered at ClinicalTrials.gov (NCT06850987). **Results:** Baseline apelin-13 levels were significantly higher in stage III–IV periodontitis and decreased in both groups after NSPT, whereas apelin-36 increased over time and was higher in stage I–II periodontitis at baseline. Apelin-13 was positively associated with clinical periodontal parameters, while apelin-36 showed negative associations. TAS remained higher in stage I–II periodontitis, whereas OSI was consistently higher in stage III–IV periodontitis. **Conclusions:** These findings suggest that the combined assessment of GCF apelin isoforms and oxidative stress markers may provide useful biomarker information for distinguishing periodontitis severity and monitoring biochemical responses to NSPT.

## 1. Introduction

Periodontal diseases are chronic and complex conditions characterized by infectious and inflammatory destruction of the supporting tissues surrounding the teeth. This process, which usually begins with gingivitis, may progress to periodontitis if left untreated, leading to irreversible losses in connective tissue attachment and alveolar bone. Although the main etiologic factor of the disease is microbial dental plaque, the host response plays a crucial role in the severity and progression of the disease [1]. Therefore, in addition to classic inflammatory mediators, studies on new biomolecules involved in the regulation of inflammation have increased in recent years [2,3,4]. Among these biomolecules, adipokines, which are cytokines derived from adipose tissue, have become an important research area in the pathogenesis of periodontal disease due to their roles in immune response, inflammation, and tissue remodeling processes [5,6]. Furthermore, changes observed in adipokine levels after periodontal therapy suggest that they may be used as biomarkers for periodontal diagnosis [5].

One of the adipokines investigated in various studies is apelin, which was identified in 1998 and exerts its effects by binding to the APJ receptor [5,7]. This peptide, secreted from adipose tissue, influences a variety of physiologic processes, including cardiovascular functions, maintenance of fluid-electrolyte balance, modulation of immune response, and regulation of oxidative stress [7]. Due to its roles in metabolic and inflammatory regulation, apelin has recently been considered a potential target molecule in studies aimed at understanding the biochemical basis of periodontitis.

Among the isoforms of apelin, apelin-13 and apelin-36 in particular play distinct biologic roles in the modulation of inflammatory responses, tissue repair, and maintenance of metabolic homeostasis [8,9]. Apelin-13 has been reported to possess high biologic activity, activating pro-inflammatory signaling pathways [10,11]. Apelin-36, on the other hand, has been shown to exhibit antioxidant properties by suppressing oxidative stress and preserving cellular homeostasis [12,13]. However, the specific effects of these isoforms in the pathogenesis of periodontal disease have not yet been fully elucidated. Serum apelin levels are elevated in systemic diseases such as obesity and diabetes. This suggests that apelin may play a role in the development and progression of periodontitis. Furthermore, it is proposed that apelin released from periodontal tissues may also contribute to the pathogenesis of these systemic diseases [14]. Previous studies have found that serum and salivary apelin levels of individuals with diabetes and periodontitis are higher compared with individuals who are systemically and periodontally healthy [15,16]. Yoldaş et al. [17] also reported that gingival crevicular fluid (GCF) apelin levels were significantly higher in individuals with periodontitis compared with those with gingivitis and periodontally healthy subjects.

Oxidative stress also plays an important role in the pathogenesis of periodontitis. Excessive production of reactive oxygen species and an insufficient antioxidant response may contribute to periodontal tissue destruction and may reflect the severity of periodontal inflammation. Total antioxidant status (TAS), total oxidant status (TOS), and oxidative stress index (OSI) provide an integrated assessment of systemic or local oxidative balance and may be useful for evaluating treatment-related changes [18,19,20,21]. However, the relationship between apelin isoforms and oxidative stress in periodontal tissues has not yet been clearly established.

To the best of our knowledge, no previous study has investigated the effects of non-surgical periodontal therapy (NSPT) on GCF apelin-13 and apelin-36 levels in systemically healthy individuals with different stages of periodontitis. Furthermore, the relationship between these apelin isoforms and oxidative stress parameters during periodontal therapy remains unclear. Therefore, the present study aimed to evaluate changes in GCF apelin-13, apelin-36, TAS, TOS, and OSI levels following NSPT in individuals with stage I–II and stage III–IV periodontitis. It was hypothesized that apelin-13, TOS, and OSI levels would be higher, whereas apelin-36 and TAS levels would be lower, in individuals with stage III–IV periodontitis. It was also hypothesized that these parameters would change following NSPT in association with improvements in clinical periodontal parameters.

## 2. Materials and Methods

Trial registration: This study was retrospectively registered at ClinicalTrials.gov (NCT06850987) on 16 February 2025. TREND Statement Checklist can be found in Supplementary Materials File S1.

### 2.1. Study Population

The protocol of this study was approved by the Non-Interventional Clinical Research Ethics Committee of Recep Tayyip Erdoğan University (Decision number: 2024/208) and was conducted in accordance with the principles of the Declaration of Helsinki, revised in 2013. The purpose and content of the study were clarified to all participants, and written informed consent was obtained. The study enrolled 61 systemically healthy individuals who presented to the Department of Periodontology, Faculty of Dentistry, Recep Tayyip Erdoğan University. The sample size was determined using the Repeated Measures ANOVA, between factors test, with G*Power software (ver. 3.1.9.7). In a two-group study, assuming 99% power (1 − β = 0.99), an effect size of 0.50 (Cohen’s f), a 5% type I error level (α = 0.05), and a repeated measures correlation (ρ) of 0.50, it was determined that a total of 48 participants were required, with at least 24 participants per group. To increase the study’s power, 61 individuals were included.

The inclusion criteria were being systemically healthy, having a diagnosis of periodontitis, having at least 20 teeth, and being aged 18–65 years. The exclusion criteria were smoking, using anti-inflammatory drugs in the last 3 months, using antibiotics/corticosteroids in the last 6 months, being pregnant or lactating, having received periodontal treatment in the last 6 months, and having systemic conditions that might affect periodontal status, such as diabetes, cardiovascular diseases, obesity, or metabolic syndrome.

Based on clinical and radiologic evaluations, and according to the 2017 World Workshop Classification of Periodontal Diseases [22], the participants were divided into two groups:

- Group 1 (G1) (n = 31): Stage I–II periodontitis (CAL ≤ 4 mm, PPD ≤ 5 mm, bone loss of 15–33% in the coronal third)
- Group 2 (G2) (n = 30): Stage III–IV periodontitis (CAL ≥ 5 mm, PPD ≥ 6 mm, bone loss extending to the apical third of the root and may include a history of tooth loss)

After periodontal measurements, all participants received non-surgical periodontal therapy (NSPT).

### 2.2. Clinical Periodontal Measurements

From all teeth, probing pocket depth (PPD), clinical attachment loss (CAL), bleeding on probing (BOP) [23], gingival index (GI) [24], and plaque index (PI) [25] were recorded. PPD and CAL were measured at six sites (mesiobuccal, distobuccal, buccal, mesiolingual, distolingual, lingual), and other indices were recorded at four sites. Measurements were performed using a Williams periodontal probe (Hu-Friedy Manufactoring Co., LLC, Chicago, IL, USA). All clinical measurements were performed by an experienced periodontist (G.N.A). The intraclass correlation coefficients (ICC) were 0.975 for PPD and 0.991 for CAL, indicating high agreement. Bone loss was evaluated using panoramic radiographs. Clinical periodontal measurements were repeated at baseline, and 1, 3, and 6 months after NSPT.

### 2.3. Collection of GCF Samples

In the morning, GCF samples were collected from each participant using PerioPaper^®^ strips (OraFlow Inc., Amityville, NY, USA) (G.N.A). GCF samples were collected from the six deepest interproximal periodontal pocket sites in each participant. At baseline, the exact tooth numbers and anatomical surfaces of these selected sites were meticulously recorded to ensure longitudinal tracking. The same pre-recorded sites were sampled at baseline and at the 1-, 3-, and 6-month follow-up visits regardless of any pocket depth reduction achieved by the therapy. During sampling, cotton rolls were used for isolation, and air-drying was applied. Strips were inserted into the pocket for 30 s. Strips contaminated with blood and saliva were excluded from the study. The GCF volume on the collected strips was measured using the Periotron 8010^®^ (Periotron 8010; Harco Electronics, Winnipeg, MB, Canada) device and then transferred into Eppendorf tubes containing 500 μL phosphate-buffered saline (PBS, pH 7.4). Sampling was performed at baseline (T0), and at 1 month (T1), 3 months (T3), and 6 months (T6) after NSPT. All samples were stored at −80 °C until analysis. The collected GCF samples were subsequently analyzed for apelin-13, apelin-36, TAS, and TOS. OSI values were calculated from TAS and TOS measurements.

### 2.4. Biochemical Analyses

All biochemical analyses were performed under standardized laboratory conditions, and the measurements were duplicated by a single, experienced biochemistry specialist who was blinded to the groups (S.M.K.).

#### 2.4.1. Apelin-13 and Apelin-36 Measurements

GCF samples were analyzed using enzyme-linked immunosorbent assay (ELISA) in the Biochemistry Laboratory of the Faculty of Medicine, Recep Tayyip Erdoğan University. Apelin-13 (BT Lab kit Bioassay Technology Laboratory, Shanghai Korain Biotech Co., Ltd., Shanghai, China, Cat. No E1273Hu) and apelin-36 (BT Lab kit Bioassay Technology Laboratory, Shanghai Korain Biotech Co., Ltd., Shanghai, China, Cat. No E2037Hu) parameters were evaluated. All procedures were performed according to the manufacturer’s protocol. After loading the samples into the microplate wells, incubation, washing, substrate addition, and reaction termination steps were followed. Optical densities were measured spectrophotometrically at 450 nm. The values were calculated using standard curves and expressed as ng/L (apelin-13) and nmol/L (apelin-36), respectively.

#### 2.4.2. Determination of TAS, TOS and OSI

TAS and TOS levels in GCF samples were determined using commercially available assay kits (Rel Assay Diagnostics, Gaziantep, Turkey) according to the manufacturer’s instructions. TAS results were expressed as mmol Trolox equivalent/L, whereas TOS results were expressed as μmol H_2_O_2_ equivalent/L. Measurements were performed using an automated analyzer (AU680, Beckman Coulter, Brea, CA, USA). The oxidative stress index (OSI) was calculated as the percentage ratio of TOS to TAS. Since TAS and TOS were expressed in different units, TAS values were first converted from mmol Trolox equivalent/L to μmol Trolox equivalent/L before OSI calculation. The following formula was used:

OSI (arbitrary units) = [TOS (μmol H_2_O_2_ equivalent/L)/TAS (μmol Trolox equivalent/L)] × 100.

### 2.5. Statistical Analysis

Data was analyzed using the SPSS software (IBM SPSS for Windows, ver. 26). For the normality of continuous variables, the Shapiro-Wilk test and skewness-kurtosis tests were used. The independent sample *t*-test was used for comparisons between two independent groups, one-way analysis of variance (ANOVA) for comparisons among more than two groups, and repeated measures ANOVA for time-dependent measurements. The assumptions of repeated-measures ANOVA were evaluated before analysis. Sphericity was assessed using Mauchly’s test, and when the assumption of sphericity was violated, the Greenhouse–Geisser correction was applied. Effect sizes were reported as partial eta squared (ηp^2^) for repeated-measures ANOVA analyses. When significant main effects were detected, pairwise comparisons were performed using the Bonferroni correction to adjust for multiple comparisons. Chi-square test was used to determine the relationships between categorical variables. Pearson correlation analysis was used to evaluate relationships between continuous variables. Correlation coefficients were classified as low (0.00–0.30), moderate (0.30–0.70), and high (0.70–1.00). A significance level of *p* < 0.05 and *p* < 0.01 was considered for all tests [26].

## 3. Results

### 3.1. Demographic and Clinical Findings

A total of 61 participants were enrolled in the study, with 31 in G1 and 30 in G2. There was no significant difference between the groups in terms of sex and age (*p* > 0.05). The mean ages were 47 ± 8.21 years in G1 and 46 ± 8.63 years in G2 (*p* > 0.05) (Table 1).

Changes in clinical parameters such as PI, GI, BOP, PPD, and CAL at baseline, T1, T3, and T6 were evaluated. At baseline, all periodontal clinical parameters were significantly worse in G2 compared with G1 (*p* < 0.05). In both groups, significant clinical improvements were observed in all parameters at T1 compared with baseline (*p* < 0.01). In the G1 group, PI values showed a slight but statistically significant increase at T3 and T6 compared with T1 (*p* < 0.01), whereas they continued to decrease in the G2 group, although this reduction was not statistically significant (*p* > 0.05). No significant differences in GI values were observed among T1, T3, and T6 in the G1 group (*p* > 0.05), while the G2 group exhibited a slight but statistically significant decrease at T3 and T6 compared with T1 (*p* < 0.01). In G2, BOP values decreased significantly at T3 and T6 compared with T1 (*p* < 0.01), and PPD, CAL, and GCF volume continued to show a significant decrease at T3 and T6 compared with T1 in both groups (*p* < 0.01) (Table 1). Repeated-measures ANOVA demonstrated significant time effects for all clinical periodontal parameters, with large effect sizes (PI: ηp^2^ = 0.705; GI: ηp^2^ = 0.715; BOP: ηp^2^ = 0.782; PPD: ηp^2^ = 0.866; CAL: ηp^2^ = 0.939; and GCF volume: ηp^2^ = 0.659; all *p* < 0.001).

### 3.2. GCF Apelin-13 and Apelin-36 Levels

At baseline, apelin-13 levels were significantly higher in G2 compared with G1 (*p* = 0.024), whereas no significant differences were observed between the groups at T1, T3, and T6 (*p* > 0.05). In within-group analyses, apelin-13 levels decreased significantly over time in both groups. At T1 and T3, a slight but non-significant decrease was observed compared with baseline, and a significant decrease was found at T6 (*p* < 0.01).

At baseline, apelin-36 levels were significantly higher in G1 compared with G2 (*p* < 0.001). No significant difference was found between the groups at T1 and T6 (*p* > 0.05). Apelin-36 levels increased over time in both groups, with the highest levels observed at T6 (*p* < 0.001) (Table 2). Repeated-measures ANOVA revealed significant time effects for apelin-13 (ηp^2^ = 0.252, *p* < 0.001) and apelin-36 (ηp^2^ = 0.260, *p* < 0.001).

### 3.3. GCF Oxidative Stress Parameters

TAS, TOS, and OSI levels in GCF samples were evaluated at T0, T1, T3, and T6 following NSPT. TAS levels were significantly higher in G1 than in G2 at all evaluation periods (*p* < 0.05). Longitudinal analysis revealed a significant decrease in TAS levels in G1 and a significant increase in G2 over time (*p* < 0.05). TOS levels were significantly lower in G1 than in G2 at T0, T1, and T3 (*p* < 0.05), whereas no significant difference was observed at T6 (*p* > 0.05). While TOS levels increased significantly over time in G1 (*p* < 0.05), no significant temporal change was observed in G2 (*p* > 0.05). OSI values were significantly higher in G2 than in G1 throughout the study period and demonstrated a significant reduction following NSPT, particularly in the advanced periodontitis group (*p* < 0.05).

These findings indicate an improvement in oxidative stress status following periodontal therapy (Table 2). Repeated-measures ANOVA revealed significant time effects for TAS (ηp^2^ = 0.251, *p* < 0.001), TOS (ηp^2^ = 0.108, *p* = 0.001), and OSI (ηp^2^ = 0.103, *p* < 0.001).

Estimated marginal means (EMMs) and their corresponding 95% confidence intervals (CIs) were calculated for each group at baseline and at the 1-, 3-, and 6-month follow-up assessments (Table 3).

### 3.4. Correlation Analyses

At baseline, apelin-13 levels showed a significant positive correlation with periodontal parameters (*p* < 0.05), whereas apelin-36 levels showed a negative correlation with clinical periodontal parameters (*p* < 0.01). At T1, T3, and T6, no statistically significant correlations were observed between apelin-13 or apelin-36 levels and clinical periodontal parameters (*p* > 0.05), despite concurrent overall improvements in both biochemical and clinical measurements following NSPT.

Regarding oxidative stress markers, no significant correlations were observed between apelin-13 and TAS, TOS, or OSI at T0, T1, or T3 (all *p* > 0.05). However, apelin-13 showed a significant positive correlation with TAS at 6 months (r = 0.336, *p* = 0.008), whereas no significant correlations were found with TOS or OSI (*p* > 0.05). In contrast, apelin-36 demonstrated a significant positive correlation with TAS (r = 0.511, *p* < 0.001) and significant negative correlations with TOS (r = −0.320, *p* = 0.012) and OSI (r = −0.370, *p* = 0.003) at baseline. At 3 months, apelin-36 was negatively correlated with TAS (r = −0.269, *p* = 0.036) and positively correlated with TOS (r = 0.352, *p* = 0.005) and OSI (r = 0.284, *p* = 0.026), whereas no significant correlation was observed at 1 or 6 months (*p* > 0.05) (Table 4).

## 4. Discussion

To the best of our knowledge, this is the first study to comparatively investigate the effects of NSPT on GCF apelin-13 and apelin-36 levels in individuals with stage I–II and stage III–IV periodontitis. The primary findings were a decrease in apelin-13 levels and an increase in apelin-36 levels following NSPT. In addition, apelin-13 showed positive correlations with clinical periodontal parameters, whereas apelin-36 demonstrated negative correlations. Furthermore, the associations between apelin-36 and oxidative stress markers (TAS, TOS, and OSI) changed over time following periodontal therapy, whereas apelin-13 exhibited only limited associations with oxidative stress parameters. Nevertheless, these findings should be interpreted within the complex and multifactorial inflammatory environment of periodontal disease rather than as evidence of a single biological pathway.

Recent studies on the roles of adipokines in chronic inflammatory diseases have shown that these molecules may influence both systemic metabolic processes and the regulation of local inflammatory responses [27,28]. In addition to studies reporting the anti-inflammatory effects of apelin [29,30], there are studies indicating elevated apelin levels in patients with metabolic syndrome and obesity [31,32,33]. Although some studies have investigated the relationship between periodontal disease and apelin, no studies have specifically examined the associations of apelin-13 and apelin-36 with periodontitis stages or their responses to NSPT.

Previous studies have shown that NSPT is effective in both early and advanced stages of periodontitis and leads to reductions in clinical periodontal parameters. Peralta et al. [34] reported significant improvements in PI, GI, PPD, and CAL following NSPT in patients with stage II, III, and IV periodontitis. Huo et al. [35] found significant improvements in periodontal parameters at 1- and 6-month follow-ups in patients with stage III–IV periodontitis. Consistent with the literature, the present study observed significant improvements in PI, GI, BOP, PPD, and CAL in both stage I–II and stage III–IV periodontitis groups after NSPT.

Apelin-13 has been shown to influence bone metabolism and the proliferation, apoptosis, and differentiation of osteoblasts, suggesting a potential role in periodontal disease [14]. In our study, apelin-13 levels were significantly higher in the stage III–IV group compared with the stage I–II group at baseline. At 6 months post-NSPT, levels significantly decreased in both groups. These results suggest that apelin-13 may be associated with the severity of periodontal inflammation. Previous studies evaluating total apelin levels also support these findings. Yoldaş et al. [17] reported that GCF apelin levels were lower in individuals with gingivitis than in patients with periodontitis, yet higher than in periodontally healthy individuals, suggesting apelin as a potential diagnostic biomarker in periodontal disease. Similarly, studies comparing serum and salivary total apelin levels in systemically and periodontally healthy individuals, patients with type 2 diabetes, and non-diabetics with periodontitis demonstrated higher apelin levels in patients with periodontitis, with the highest levels observed in individuals with both diabetes and periodontitis [15,16,36]. Since these studies did not distinguish between specific apelin isoforms, direct comparison with the present findings should be interpreted cautiously. Unlike previous studies evaluating total apelin concentrations, the present study separately analyzed apelin-13 and apelin-36 levels, revealing distinct associations between these isoforms and periodontal inflammation.

In our study, baseline apelin-13 levels positively correlated with PI, GI, BOP, PPD, and CAL. Similarly, Yoldaş et al. [17] reported positive correlations between GCF apelin levels and clinical periodontal parameters. It has also been reported that the application of apelin together with *Fusobacterium nucleatum* increases pro-inflammatory molecules in periodontal ligament cells, suggesting that apelin may mediate the deleterious effects of obesity on periodontal tissues [37]. In contrast, an in vitro study by Lee et al. [38] demonstrated that apelin-13 suppressed tumor necrosis factor-alpha (TNF-α)-induced inflammatory gene expression, indicating potential anti-inflammatory effects of this isoform under certain experimental conditions. Taken together, these findings indicate that apelin-13 may exhibit both pro-inflammatory and anti-inflammatory properties in the pathogenesis of periodontitis, depending on the local inflammatory environment and the specific biological conditions investigated.

Apelin-36 is known to play a role in key physiologic processes such as metabolic regulation, inflammation, and angiogenesis [39]. In our study, apelin-36 levels significantly increased after NSPT. At baseline, levels were higher in stage I–II periodontitis than in stage III–IV, whereas at 1- and 3-month post-NSPT follow-ups, although not significantly, levels were higher in stage III–IV. The negative correlation of apelin-36 with clinical periodontal parameters at baseline together with its increase following NSPT, suggest an inverse association with periodontal inflammatory severity. However, these findings do not establish a causal or protective role for apelin-36 in periodontal healing.

Although no previous study has specifically investigated apelin-36 in periodontal disease, experimental studies conducted in other inflammatory conditions have reported anti-inflammatory and antioxidant effects associated with this isoform [40,41,42,43]. Therefore, the distinct increase observed in apelin-36 following NSPT might reflect a potential link to periodontal healing through the reduction of inflammation, promotion of tissue repair, and attenuation of oxidative stress. Conversely, increases in apelin-36 levels have also been associated with inflammatory conditions such as metabolic syndrome [31,33] and obesity [31,32]. In the present study, the increase in apelin-36 levels following NSPT, together with overall clinical improvements, suggests a possible association with periodontal healing and tissue repair, further supporting its alignment biologic changes accompanying periodontal healing and resolution of inflammation.

Interestingly, baseline TAS levels were significantly higher in the stage I–II periodontitis group than in the stage III–IV group. This finding may indicate differences in antioxidant response capacity according to disease severity. It is possible that prolonged oxidative stress in advanced periodontitis may contribute to impairment of local antioxidant defense mechanisms [44]. In the stage I–II group, TAS levels showed a significant time-dependent fluctuation, characterized by an initial reduction at 1 month followed by a partial increase at 3 months and a slight decrease at 6 months. These variations should be interpreted cautiously, as they may reflect dynamic changes in local antioxidant responses during different phases of periodontal healing rather than a uniform deterioration or improvement in antioxidant capacity. Similar findings have been reported in previous studies indicating that antioxidant activity may decrease after periodontal therapy as inflammatory and oxidative stimuli are reduced [20,45,46].

Regarding TOS, levels were significantly higher in the stage III–IV periodontitis group than in the stage I–II group at baseline and at the 1- and 3-month follow-ups, whereas the intergroup difference was no longer significant at 6 months. TOS levels increased significantly over time in the stage I–II group, with the highest value observed at 6 months, whereas no significant temporal change was detected in the stage III–IV group. OSI values were significantly higher in the stage III–IV group at all evaluation time points. OSI decreased significantly at 6 months in the stage III–IV group, whereas a comparable improvement was not observed in the stage I–II group, in which OSI increased over time. Taken together, NSPT did not produce a uniform reduction in local oxidative stress across both groups; instead, changes in TOS and OSI varied according to disease severity and follow-up time.

Experimental evidence indicates that the apelin/APJ system may influence redox homeostasis by regulating ROS production, lipid peroxidation, mitochondrial function, and endogenous antioxidant defense mechanisms. However, the direction and magnitude of these effects appear to depend on the cellular and pathological context, as apelin has been reported to exert both antioxidant and pro-oxidant effects under different experimental conditions [12,47,48].

In this context, the different patterns of change observed in TOS and OSI in the present study suggest that there is no linear relationship between clinical periodontal improvement and reduction in oxidative stress; rather, local redox balance appears to exhibit a dynamic response that is sensitive to environmental conditions. Nevertheless, because the available mechanistic evidence is largely derived from non-periodontal experimental models, these findings should be interpreted cautiously; a causal contribution of apelin to the observed redox changes cannot be inferred from the present study.

An important finding of the present study was the association between apelin isoforms and oxidative stress parameters. At baseline, apelin-36 showed a significant positive correlation with TAS and significant negative correlations with TOS and OSI, supporting a potential association between this isoform and oxidative stress regulation. However, these correlations were not consistently maintained throughout the follow-up period, indicating that the relationship between apelin and oxidative stress may change during different phases of periodontal healing. Furthermore, apelin-36 levels increased following NSPT, coinciding with improvements in periodontal status and oxidative stress balance. These findings suggest that apelin-36 may be involved in antioxidant defense mechanisms and tissue repair processes during periodontal healing. Since apelin expression and function, reactive oxygen species generation, and antioxidant capacity may vary at different rates during these stages [49,50,51], the associations may be transiently disrupted or even shift direction [21,50]. Accordingly, the inverse relationship between apelin-36 and TAS, and the positive relationship with TOS and OSI at the 3rd month, may represent a transient, accelerated increase in apelin-36 in response to residual or fluctuating oxidative activity during tissue remodeling, rather than a direct pro-oxidant effect [50,52]. Previous experimental studies have reported that apelin-36 may suppress oxidative stress by reducing lipid peroxidation and enhancing antioxidant enzyme activity [12,42,43]. Consistent with these observations, the present findings indicate that alterations in apelin-36 levels occur in parallel with changes in oxidative stress markers following periodontal therapy, supporting a potential role for this isoform in the regulation of redox homeostasis.

This study has some limitations. Owing to its relatively limited sample size, the findings may not be directly generalizable to the broader population. Although apelin isoforms and oxidative stress parameters were evaluated, additional inflammatory cytokines and host-response biomarkers were not assessed. Periodontitis is a multifactorial inflammatory disease involving numerous cytokines, adipokines, oxidative stress mediators, and host-response pathways. Therefore, the present findings should not be interpreted as indicating that apelin-13 or apelin-36 alone can explain periodontal disease progression or healing. Despite our strict eligibility criteria, unmeasured factors such as individual dietary habits, psychological stress, and sleep patterns might have subtly influenced the biochemical findings, representing as is an inherent limitation of biomarker research. Furthermore, a major limitation of this study is the absence of a periodontally healthy control group. The absence of a healthy baseline cohort limited our ability to definitively determine whether the reported biomarker levels reflected disease-specific changes or simply normal biological variation within the population, thus preventing the establishment of reliable reference levels for the biomarkers examined. The exclusion of smokers and individuals with obesity or metabolic disease from the study may have limited the generalizability of the results to larger periodontitis populations where these characteristics are common. Therefore, the present findings should be interpreted primarily as differences between periodontitis severity groups and as longitudinal biochemical changes following NSPT rather than as differences between periodontal health and disease or as definitive diagnostic reference values. To more clearly define the biomarker relevance of apelin isoforms in periodontal disease, future studies should include gingivitis and periodontally healthy groups, larger sample sizes, longer follow-up periods, and analyses of additional parameters such as serum and saliva.

## 5. Conclusions

Within the limitations of this study, NSPT significantly affected both apelin isoforms and oxidative stress parameters in GCF. Apelin-13 appeared to be mainly associated with periodontal inflammatory burden, whereas apelin-36 showed a more consistent correlation with oxidative stress markers, particularly at baseline. These findings suggest that the combined assessment of apelin isoforms and oxidative stress markers may provide additional insight into periodontal disease activity and treatment response. Further longitudinal studies including periodontally healthy individuals and additional inflammatory biomarkers are required to clarify the biologic role of apelin in periodontal disease. However, while these findings suggest that apelin isoforms carry potential as biomarkers, additional validation studies with larger and more diverse populations are still required before any definitive clinical application can be considered.

## Figures and Tables

**Table 1:** Demographic characteristics, clinical periodontal parameters and gingival crevicular fluid during the follow-up period.

	G1 (n = 31)					G2 (n = 30)					Intergroup Difference p***			
	Baseline	1 Month	3 Months	6 Months	p*	Baseline	1 Month	3 Months	6 Months	p**	Baseline	1 Month	3 Months	6 Months
Age (years)	47 ± 8.21					46 ± 8.63					0.472			
Sex (male/female)	18/13					17/13					0.912			
Plaque Index	0.9 ± 0.38 ^C,a^	0.22 ± 0.22 ^A,a^	0.35 ± 0.3 ^B,a^	0.42 ± 0.33 ^B^	0.01	1.9 ± 0.52 ^B,b^	0.74 ± 0.45 ^A,b^	0.56 ± 0.41 ^A,b^	0.55 ± 0.52 ^A^	0.01	0.01 **	0.01 **	0.024 *	0.248
Gingival Index	0.87 ± 0.36 ^B,a^	0.22 ± 0.31 ^A,a^	0.21 ± 0.29 ^A,a^	0.26 ± 0.33 ^A,a^	0.01	1.74 ± 0.45 ^C,b^	0.87 ± 0.47 ^B,b^	0.6 ± 0.43 ^A,b^	0.47 ± 0.46 ^A,b^	0.01	0.01 **	0.01 **	0.01 **	0.04 *
BOP (%)	48.16 ± 17.67 ^B,a^	9.35 ± 10.36 ^A,a^	14.39 ± 16.25 ^A,a^	13.19 ± 15.7 ^A^	0.01	91.4 ± 12.03 ^D,b^	41.67 ± 21.31 ^C,b^	31.1 ± 24.58 ^B,b^	18.93 ± 21.8 ^A^	0.01	0.01 **	0.01 **	0.003 **	0.242
PPD (mm)	2.45 ± 0.32 ^D,a^	1.69 ± 0.24 ^C,a^	1.24 ± 0.18 ^B,a^	1.13 ± 0.2 ^A,a^	0.01	3.76 ± 1.06 ^D,b^	3.04 ± 1.04 ^C,b^	2.39 ± 1.03 ^B,b^	2 ± 0.91 ^A,b^	0.01	0.01 **	0.01 **	0.01 **	0.01 **
CAL (mm)	3.42 ± 0.42 ^D,a^	2.45 ± 0.37 ^C,a^	1.55 ± 0.41 ^B,a^	1.16 ± 0.42 ^A,a^	0.01	5.19 ± 0.97 ^D,b^	4.19 ± 1 ^C,b^	3.19 ± 1.05 ^B,b^	2.41 ± 0.89 ^A,b^	0.01	0.01 **	0.01 **	0.01 **	0.01 **
GCF (μL)	38.8 ± 10.67 ^C,a^	27.22 ± 8.05 ^B,a^	22.37 ± 7.13 ^A,a^	21 ± 6.93 ^A^	0.01	46.36 ± 11.32 ^C,b^	33.46 ± 8.62 ^B,b^	26.51 ± 8.21 ^A,b^	23.3 ± 8.01 ^A^	0.01	0.009 **	0.005 **	0.04 *	0.234

Data are presented as mean (±standard deviation). Independent samples *t*-test was used for comparisons between two independent groups, repeated measures ANOVA was performed to analyze time-dependent changes, and Chi-square test was used for categorical variable. Greenhouse–Geisser corrected *p*-values are reported when the assumption of sphericity was violated. *p*-values < 0.05 (*) and <0.01 (**) were considered statistically significant. BOP: Bleeding on probing, PPD: Probing pocket depth, CAL: Clinical attachment loss, GCF: Gingival crevicular fluid, G1: Systemically healthy Stage I-II periodontitis group; G2: Systemically healthy Stage III-IV periodontitis group, Different superscript uppercase letters (A, B, C, D) indicate statistically significant differences within groups over time. Different superscript lowercase letters (a, b) indicate statistically significant differences between groups at the same time point. mm: millimeter, μL: microliter, %: percent, p*: Significant time-dependent change within the G1, p**: Significant time-dependent change within the G2, p***: Significant time-dependent change between groups.

**Table 2:** Apelin isoforms and oxidative stress markers during the follow-up period.

	G1 (n = 31)					G2 (n = 30)					Intergroup Difference p***			
	Baseline	1 Month	3 Months	6 Months	p*	Baseline	1 Month	3 Months	6 Months	p**	Baseline	1 Month	3 Months	6 Months
Apelin-13 (ng/L)	37.78 ± 6.99 ^B,a^	35.34 ± 10.56 ^B^	37.61 ± 10.64 ^B^	29.36 ± 5.29 ^A^	0.001	43.76 ± 12.35 ^B,b^	36.71 ± 7.14 ^B^	40.09 ± 7.5 ^B^	28.9 ± 4.96 ^A^	0.001	0.024 *	0.555	0.296	0.729
Apelin-36 (nmol/L)	57.86 ± 5.52 ^A,a^	62.32 ± 8.21 ^B^	61.34 ± 8.43 ^B,a^	74.24 ± 9.99 ^C^	0.001 **	46.36 ± 9.51 ^A,b^	64.03 ± 7.85 ^B^	66.87 ± 8.07 ^B,b^	72.3 ± 5.92 ^C^	0.001 **	0.001 **	0.411	0.011 *	0.356
TAS (mmol/L)	0.164 ± 0.03 ^B,a^	0.146 ± 0.02 ^A,a^	0.155 ± 0.02 ^B,a^	0.15 ± 0.02 ^AB,a^	0.005 **	0.04 ± 0.03 ^A,b^	0.05 ± 0.04 ^A,b^	0.04 ± 0.02 ^A,b^	0.11 ± 0.04 ^B,b^	0.001 **	0.001 **	0.001 **	0.001 **	0.001 **
TOS (µmol/L)	0.15 ± 0.09 ^AB,a^	0.17 ± 0.05 ^B,a^	0.13 ± 0.05 ^A,a^	0.2 ± 0.06 ^C^	0.001 **	0.19 ± 0.07 ^b^	0.23 ± 0.04 ^b^	0.2 ± 0.06 ^b^	0.21 ± 0.07	0.167	0.025 *	0.001 **	0.001 **	0.597
OSI	91.17 ± 56.5 ^A,a^	121.87 ± 37.74 ^B,a^	84.83 ± 33.4 ^A,a^	135.12 ± 37.03 ^C,a^	0.001 **	702.5 ± 620 ^B,b^	734.39 ± 595.7 ^B,b^	615.9 ± 333.7 ^B,b^	235.43 ± 153 ^A,b^	0.001 **	0.001 **	0.001 **	0.001 **	0.001 **

Data are presented as mean (±standard deviation). Independent samples *t*-test was used for comparisons between two independent groups, and repeated measures ANOVA was performed to analyze time-dependent changes. Greenhouse–Geisser corrected *p*-values are reported when the assumption of sphericity was violated. *p*-values < 0.05 (*) and <0.01 (**) were considered statistically significant. TAS: Total antioxidant status; TOS: Total oxidant status; OSI: Oxidative stress index, G1: Systemically healthy Stage I-II periodontitis group; G2: Systemically healthy Stage III-IV periodontitis group, Different superscript uppercase letters (A, B, C) indicate statistically significant differences within groups over time. Different superscript lowercase letters (a, b) indicate statistically significant differences between groups at the same time point. ng: nanogram, L: liter, nmol: nanomole µmol: micromole, mmol: milimole, %: percent, p*: Significant time-dependent change within the G1, p**: Significant time-dependent change within the G2, p***: Significant time-dependent change between groups.

**Table 3:** Estimated marginal means and 95% confidence intervals according to assessment time.

Parameter	%95 CI			
	Baseline (Lower–Upper)	1 Month (Lower–Upper)	3 Months (Lower–Upper)	6 Months (Lower–Upper)
Plaque Index	1.266–1.501	0.378–0.562	0.360–0.545	0.374–0.596
Gingival Index	1.188–1.400	0.432–0.637	0.308–0.495	0.256–0.461
BOP (%)	64.967–73.198	20.521–29.468	17.171–27.760	11.099–20.854
PPD (mm)	2.887–3.302	2.151–2.551	1.605–1.994	1.378–1.726
CAL (mm)	4.075–4.475	3.093–3.494	2.133–2.556	1.582–1.943
GCF (μL)	39.689–45.200	28.136–32.320	22.403–26.350	20.192–24.033
Apelin-13 (ng/L)	38.187–43.351	33.724–38.329	36.494–41.199	27.819–30.446
Apelin-36 (nmol/L)	50.113–54.115	61.116–65.232	61.989–66.218	71.176–75.367
TAS (mmol/L)	0.095–0.109	0.092–0.109	0.093–0.102	0.121–0.138
TOS (µmol/L)	0.151–0.192	0.189–0.212	0.153–0.179	0.189–0.221
OSI	283.145–510.506	319.070–537.185	289.102–411.659	156.536–214.012

BOP: Bleeding on probing, PPD: Probing pocket depth, CAL: Clinical atachment loss, GCF: Gingival crevicular fluid, %: percentage, mm: milimeter, ng: nanogram, L: liter, nmol: nanomole, μL: microlitre, TAS: Total antioxidant status, TOS: Total oxidant status, OSI: Oxidative stress index, CI: confidence interval.

**Table 4:** Time-dependent correlations between apelin isoforms, clinical periodontal parameters, and oxidative stress markers during the follow-up period.

		Baseline		1 Month		3 Months		6 Months	
		Apelin-13 (ng/L)	Apelin-36 (nmol/L)	Apelin-13 (ng/L)	Apelin-36 (nmol/L)	Apelin-13 (ng/L)	Apelin-36 (nmol/L)	Apelin-13 (ng/L)	Apelin-36 (nmol/L)
Plaque Index	r	0.354 **	−0.397 **	0.185	0.223	0.094	0.014	−0.173	0.185
	*p*	0.005	0.002	0.153	0.083	0.473	0.917	0.182	0.152
Gingival Index	r	0.287 *	−0.489 **	0.165	0.229	−0.019	0.172	−0.093	0.157
	*p*	0.025	0.01	0.204	0.076	0.883	0.184	0.478	0.228
BOP (%)	r	0.326 *	−0.457 **	0.2	0.061	0.038	0.014	−0.087	0.191
	*p*	0.01	0.01	0.122	0.64	0.774	0.917	0.506	0.141
PPD (mm)	r	0.340 **	−0.443 **	0.235	0.19	0.001	0.164	−0.013	−0.122
	*p*	0.007	0.01	0.068	0.143	0.995	0.212	0.92	0.35
CAL (mm)	r	0.339 **	−0.444 **	0.218	0.174	0.014	0.218	0.015	−0.077
	*p*	0.008	0.01	0.091	0.181	0.913	0.092	0.906	0.555
GCF (μL)	r	0.129	−0.133	0.23	0.259 *	−0.006	0.133	0.164	0.168
	*p*	0.321	0.306	0.075	0.044	0.966	0.306	0.206	0.195
TAS (mmol/L)	r	−0.187	0.511 **	−0.083	−0.137	−0.162	−0.269 *	0.336 **	0.099
	*p*	0.148	0.01	0.524	0.293	0.212	0.036	0.008	0.447
TOS (µmol/L)	r	−0.196	−0.320 *	0.124	−0.044	0.115	0.352 **	0.11	0.143
	*p*	0.129	0.012	0.342	0.738	0.378	0.005	0.397	0.272
OSI	r	−0.159	−0.370 **	0.15	0.066	0.119	0.284 *	−0.181	−0.017
	*p*	0.22	0.003	0.248	0.614	0.362	0.026	0.163	0.895

Relationships between continuous variables were evaluated using Pearson correlation analysis. Statistical significance was set at *p* < 0.05 and *p* < 0.01. BOP: Bleeding on probing, PPD: Probing pocket depth, CAL: Clinical attachment loss, GCF: Gingival crevicular fluid, TAS: Total antioxidant status, TOS: Total oxidant status, OSI: Oxidative stress index, mm: millimeter, ng: nanogram, L: liter, μL: microliter, nmol: nanomole, µmol: micromole, mmol: milimole, %: percent, r: correlation coefficient, *: *p* < 0.05, **: *p* < 0.01.

## Data Availability

The data that support the findings of this study are available from the corresponding author upon reasonable request.

## Supplementary Materials

The following supporting information can be downloaded at: https://www.mdpi.com/article/10.3390/diagnostics16152484/s1, Supplementary Materials File S1: TREND Statement Checklist. Reference [53] is cited in the Supplementary Materials.

## Abbreviations

The following abbreviations are used in this manuscript:

ANOVA	Analysis of variance
APJ	Apelin receptor
BOP	Bleeding on probing
CAL	Clinical attachment loss
ELISA	Enzyme-linked immunosorbent assay
G1	Stage I–II periodontitis group
G2	Stage III–IV periodontitis group
GCF	Gingival crevicular fluid
GI	Gingival index
ICC	Intraclass correlation coefficient
L	liter
mm	millimeter
ng	nanogram
nmol	nanomole
NSPT	Non-surgical periodontal therapy
OSI	Oxidative stress index
PBS	Phosphate-buffered saline
PI	Plaque index
PPD	Probing pocket depth
ROS	Reactive oxygen species
SD	Standard deviation
T0	Baseline
T1	1 month after NSPT
T3	3 months after NSPT
T6	6 months after NSPT
TAS	Total antioxidant status
TOS	Total oxidant status
TNF-α	Tumor necrosis factor-alpha
μL	microliter
